# University of Maryland—Dental Department

**Published:** 1892-04

**Authors:** 


					University of Maryland?Dental Departmejtt.-
?The
-annual commencement exercises of the Department of Dental
Surgery of the University of Maryland were held at the Lyceum
Theater, Baltimore, Md., on Thursday, March 17, 1892.
The mandamus was read by the dean, Ferdinand J. S.
Gorgas, M.D., D.D.S.;the address to the graduates was delivered
by Rev. William T. Roberts, of Virginia, and the class oration
by H. Janney Nichols, D.D.S. The number of matriculates for
the session was one hundred and twenty-seven.
The degree of D.D.S. was conferred on the following gradu-
ates by Hon. S. Teackle "VVallis, LL. D., provost of the uni-
versity :
W. Wolsley Alton, Canada; Walter C. Anderson, Virginia;
Fletcher G. Asbill, South Carolina; Dabney G. Barnitz, Vir-
ginia; Charles F. Baylis, New York; John C.C. Beale, Maryland;
Alexander J. Beville, Texas; Samuel E. Braendle, Canada;
Winfield S. Burd, Pennsylvania; Andrew S. Burke, Penn-
sylvania; W. Bolivar Byers, South Carolina; E. M. Copenhaver,
Virginia; W. Felton Deekens, Virginia; J. Harry Deems, Jr.,
Maryland; William W. Dennis, Georgia; John H. Diddle, West
Virginia; William E. Dobson, New York; John L. Doremus,
France; Eben B. Edgers, Vermont; Robert W. Eicholtz, Penn-
sylvania; Louis Ewig, Switzerland; C. Dixie Farriss, Georgia;
Lawrence S. Fox, North Carolina; Edwin J. Gill, North
?Carolina; Eli H. Glasscock, Missouri; Geo. H. Hargrove, South
Carolina; Oscar J. Harmon, New Hampshire; Lewis E. Hess,
Maryland; Frederick C. Humberg, Maryland; Hugh B. Hutchi-
son, Virginia; Benj. L. Jefferson, Georgia; Silas J. Johnson,
Virginia; B. Arthur Jordan, California; James M. King,Canada;
C. Rogers LeFevre, Maryland; J. Clinton Macomber, Penn-
sylvania; Thomas R. Marshall, Virginia; Anthony H. Mathieu,
.Maryland; W. Glenn McGee, South Carolina; George A. Mc-
Editorial, &c. 573
Gee, South Carolina; George A. McHarg, Canada; Robert J.
McHarg, Canada; J. Morton Mcllvain, Maryland; C.Aug.Mitch-
ell, New York; Harry B. Mitchell, New York; H. Janney
Nichols, Virginia; Clyde S. Payne, California; George C.Probst,.
South Carolina; George B. Quinlan, New York; Turner A.
Ramey, AVest Virginia; Joseph L. Rathie, Virginia; E.
Reynolds, New York; Jacob Kiser, Iowa; Edmund D. Shaw,
New York; James W. Simpson, Virginia; Will R. Simpson,
South Carolina; Harry B. Smith, Bermuda; Charles B. Stouf-
fer, Pennsylvania; M. Emmert Stover, Pennsylvania; Arthur
0. Thomas, South Carolina; William A. Thrush, Illinois; Die
P. Tipton, Nebraska; Arminius W. Totten, North Carolina; W.
H. Van Nostrand, New York; Harry Van Tassell,South Dakota;
Joseph M. Veza, Austria; Frank Von Wachter, Maryland; J.
Willie Watson, West Virginia; Montgomery L. White, Texas;
Charles G. Wiley, Pennsylvania; Henry A. Wilson, New Hamp-
shire; A". W. Woodward, Virginia, J. Harvey Wool, Virgininia.

				

